# Oxidative stress and premature senescence in corneal endothelium following penetrating keratoplasty in an animal model

**DOI:** 10.1186/s12886-016-0192-6

**Published:** 2016-02-02

**Authors:** Xiaowen Zhao, Ye Wang, Yao Wang, Suxia Li, Peng Chen

**Affiliations:** State Key Laboratory Cultivation Base, Shandong Provincial Key Laboratory of Ophthalmology, Shandong Eye Institute, Shandong Academy of medical Sciences, No. 5 Yanerdao Rd, Qingdao, 266071 China; Current affiliation: Central Laboratory of the Second Affiliated Hospital, Medical College of Qingdao University, Qingdao, 266042 China

**Keywords:** Oxidative stress, Premature senescence, Corneal endothelium, Penetrating keratoplasty

## Abstract

**Background:**

The purpose of this study was to address the question of how the premature senescence process may affect corneal endothelium after penetrating keratoplasty, because the quality of donor corneal endothelial cells is important for corneal transplant success.

**Methods:**

The cell senescence and induced oxidative stress in corneal endothelium were assessed using a normal-risk orthotopic mice corneal transplantation model. Senescence associated beta-galactosidase (SA-beta-Gal) staining was used to evaluate premature senescence in the endothelium of corneal allografts. Oxidative Stress and Antioxidant Defense RT^2^-PCR Arrays and *in vitro* experimental model using H_2_O_2_ treatment were used to investigate the possible mechanism.

**Results:**

SA-beta-Gal positivity was observed obviously in mice corneal endothelium of allogenic group and the levels of p16^INK4a^ message and protein increased in endothelium of allogenic group compared to syngenic group. By PCR array, an oxidant-antioxidant imbalance was found in the endothelium of corneal allograft after PKP. The results from mice model were validated using human endothelium samples of corneal allograft after PKP. We also developed an *in vitro* experimental model using H_2_O_2_ treatment to simulate a state of oxidative stress in cultured human corneal endothelial cells (HCECs) and found that elevated ROS levels, the up-regulation of CDK inhibitors and ROS-mediated p16^INK4A^ up-regulation in HCECs occur via the ASK1-p38 MAPK pathway.

**Conclusions:**

Our results demonstrate the presence of oxidative stress and premature senescence in the endothelium of corneal allografts following PKP.

**Electronic supplementary material:**

The online version of this article (doi:10.1186/s12886-016-0192-6) contains supplementary material, which is available to authorized users.

## Background

Human corneal endothelial cells (HCECs) form a monolayer with limited regenerative potential, and these cells maintain stromal dehydration via an ion pump mechanism [1]. For the past 50 years, penetrating keratoplasty (PKP) has been the standard treatment for corneal endothelial specific dysfunctional diseases [2]. Rather than replacing the entire cornea, endothelial keratoplasty (EK) replaces the patient's endothelium with a transplanted disc of posterior stroma/Descemets/endothelium (DSEK) or Descemets/endothelium (DMEK) [3]. This relatively new procedure has revolutionized treatment of disorders of the endothelium. Additionally, lamellar keratoplasty (LKP) replaces only the diseased Bowman’s layer and the anterior, or upper part of the corneal stroma with donor material. The quality of donor corneal endothelial cells is very important for corneal transplant success. It was reported that the status of donor endothelial cells may be a necessary condition for graft transparency and long-term survival [4, 5]. Endothelial cell loss can lead to corneal opacity over time after PKP and is substantial 5 years postkeratoplasty [6]. However, there is currently little understanding of the mechanisms of the accelerated postoperative loss of endothelial cells.

DNA breaks and oxidative lesions caused by environmental insults, genetic defects, or endogenous processes belong to certain types of DNA damage. One of the critical effects of oxidative stress caused by reactive oxygen species is the induction of cellular senescence [7, 8]. Numerous studies have reported that premature senescence is closely related to organ transplant [9–12], such as renal transplantation [13]. Cellular senescence is a state of irreversible growth arrest. It can be triggered by many kinds of oncogenic or stressful stimuli including telomere shortening, the epigenetic derepression of the *INK4a/ARF* locus [8, 14]. Studies with clinical outcome of HCECs showed the exhibition signs of oxidative DNA damage and that oxidative stress affects the proliferative capacity of HCECs [15, 16]. These authors also observed that, with respect to the senescence of corneal endothelial cells, age-related relative proliferative capacity and senescence characteristics are not due to replicative senescence caused by critically short telomeres *in vitro* [17].

Studies have indicated that protein kinase activities are redox-sensitive because key cysteine residues in these proteins can undergo post-translational modifications by oxidants [18]. Several signal transduction pathways have been implicated in cell senescence and cell death, including p38 MAPK pathway. Moreover, apoptosis signal-regulating kinase 1 (ASK1) is a key element in the mechanism of stress-induced cell senescence [19]. It was reported that stress-activated ASK1 accelerates endothelial cell senescence in patients with diabetes [20] and that the inhibition of the ASK1-p38 MAPK pathway could be useful for preventing vascular ageing and for treating neurodegenerative and cardiac diseases [18, 21]. However, whether ASK1-p38 MAPK pathway underlying the cell premature senescence in pathogenesis of corneal endothelium after PKP are not well understood.

In this study, we assessed premature senescence and induced oxidative stress in corneal endothelium using a normal-risk orthotopic mice corneal transplantation model. Then, using an oxidative stress and antioxidant defense PCR array, an oxidant-antioxidant imbalance was found to be involved in the endothelium of corneal allograft after PKP. Next, we validated our results from the mice model using human endothelium samples of corneal allograft after PKP. We also developed an *in vitro* experimental model using H_2_O_2_ treatment to simulate a state of oxidative stress in cultured HCECs and found that elevated ROS levels, the up-regulation of CDK inhibitors and ROS-mediated p16^INK4A^ up-regulation in HCECs occur via the ASK1-p38 MAPK pathway.

## Methods

### Animals and the normal-risk orthotopic corneal transplantation

The study was approved by the Institutional Animal Care Committee of Shandong Eye Institute, and all of the procedures were performed according to the Association for Research and Vision in Ophthalmology (ARVO) Statement for the Use of Animals in Ophthalmic and Vision Research. Six- to eight-week-old adult BALB/c (H-2d) mice and C57BL/6 (H-2b) mice, weighing 18 to 20 g, were obtained from the Institute of Zoology Chinese Academy of Sciences. The mice were divided into two groups, syngenic groups and allogenic, each containing 20 mice. The corneal transplantations were performed as previously described [22]. Male C57BL/6 mice were used as donors, and same-aged male BALB/c mice were used as recipients. The outcomes of the procedure were compared between syngeneic grafts for which male BALB/c mice were used as both the donors and recipients. Immunosuppressive drugs were not used, either topically or systemically. Only the right eye of each mouse was used for the corneal transplantation; the left eye was undisturbed.

The corneal grafts were collected 4.5 months post-grafting, and the corneal endothelium samples were separated from the grafts. For the PCR Array analysis (SABiosciences Corp., Frederick, MD), the samples were pooled into 6 groups (SP1, SP2, SP3, AP1, AP2, and AP3). Each group comprised three corneal endothelium samples. For the Western blot analyses, the samples were pooled into 6 groups (SW1, SW2, SW3, AW1, AW2, and AW3). Each group comprised two corneal endothelium samples.

### Clinical evaluation of grafted mouse corneas, dual staining of corneal endothelium with trypan blue and alizarin red S, and immunohistochemistry

The mouse corneal grafts were examined once per week for two weeks with a slit-lamp biomicroscope (Haag-Streit model BQ-900, Switzerland). Graft opacity was scored using a scale of one to five, as previously described [22]. The corneal grafts were considered to be failures after receiving two successive scores of 3. All of the examinations were performed by two blinded observers. The corneal endothelium was examined by dual staining of the corneal endothelium with trypan blue and alizarin red S (Sigma-Aldrich, Shanghai, China) [23]. For the immunohistochemical analysis of 8-hydroxydeoxyguanosine (8-OHdG) (AB5830; Millipore, Bedford, MA), the corneal samples were fixed in cold methanol and the samples were subjected to staining using the EliVision™ plus kit (Maxim Corp, Fuzhou, China), according to the manufacturer’s protocol, and observed using a Fluorescence E800 microscope (Nikon, Tokyo, Japan).

### Real time PCR-based array analysis

Total RNA of mouse corneal endothelium was isolated using NucleoSpin RNA II System (Macherey-Nagel, Düren, Germany) according to the manufacturer’s instructions. The first-strand cDNA was synthesized from equal amounts of total RNA (1 μg/μl) using a PrimeScript® 1st Strand cDNA Synthesis Kit (TaKaRa, Dalian, China). The real-time PCR array and data analyses were performed using a RT^2^ Profiler™ PCR Array Mouse Oxidative Stress and Antioxidant Defense PCR Array (PAMM-065A, SABiosciences Corp., Frederick, MD).

### Patient tissue sample collection

After obtaining approval from the Shandong Eye Institute ethics committee and according to the tenets of the Declaration of Helsinki, between December 2005 and December 2010, 15 fresh dysfunctional corneal buttons (7.25 to 8 mm diameter) were collected prospectively from patients with failed corneal transplants. After further sample preparation, five samples were selected for analysis as shown in Table 1. The prospectively enrolled patients from which dysfunctional corneal buttons were obtained provided written informed consent.

**Table 1:** The information of all the patients

Patient No.	Age/ Gender	Affected Eye	History of Ocular Diseases	Time^a^ (years)	Experiment
1	41/M	OS	Keratoplasty for corneal opacity	5	SA-β-Gal staining
2	35/M	OS	Keratoplasty for corneal opacity	8.5	SA-β-Gal staining
3	62/M	OD	Keratoplastyfor bullous keratopathy	7	gene expression profiling
4	62/M	OD	Keratoplasty for corneal opacity	7	gene expression profiling
5	21/M	OS	Ocular trauma	7	gene expression profiling

F = female; M = male

^a^years between the first and the second corneal transplantation

Sex-matched normal human cornea samples from organ donors were provided by the International Federation of Eye Banks at the Eye Bank of Shandong, China (Qingdao, China) and were used as controls. Three samples were used for the SA-β-Gal staining (age 21, 25, and 19 years) and four samples were used for the gene expression profiling (age 21, 27, 30 and 31 years). Another five samples (three for 22 years old and two for 25 years old) were used to the HECEs culture. Corneal rims, residual parts dissected from donor corneas by a circular trephine in penetrating keratoplasty, were immediately collected for this study as shown in Table 2. All samples were maintained in corneal storage medium (Optisol; Chiron Ophthalmics, Inc., Irvine, CA) at 4 °C until immediately before the experiment.

**Table 2:** Donor Information

Age (y)	Hours^a^	Days^b^	Cause of Death	Experiment	Samples
19	4	1	Motor vehicle accident	SA-β-Gal staining	whole cornea
21	5	2	Cardiac arrest	SA-β-Gal staining	whole cornea
25	4	2	Traumatic injury	SA-β-Gal staining	whole cornea
21	6	2	Motor vehicle accident	gene expression profiling	whole corneal endothelium
27	7	1	Motor vehicle accident	gene expression profiling	whole corneal endothelium
30	6	1	Motor vehicle accident	gene expression profiling	whole corneal endothelium
31	5	2	Motor vehicle accident	gene expression profiling	whole corneal endothelium
22	4	2	Cardiac arrest	HECEs culture	corneal rims
22	1	1	Cardiac arrest	HECEs culture	corneal rims
22	2	1	Motor vehicle accident	HECEs culture	corneal rims
25	2	1	Cardiac arrest	HECEs culture	corneal rims
25	2	1	Motor vehicle accident	HECEs culture	corneal rims

^a^Number of hours between death and corneal preservation

^b^Number of days of corneal preservation in corneal storage medium at 4 °C

### RNA extraction and gene expression profiling study

Total RNA was isolated using the NucleoSpin RNA II System (Macherey-Nagel, Duren, Germany), and first-strand cDNA was synthesized using a MMLV Reverse Transcriptase 1st-Strand cDNA Synthesis Kit (Epicentre Biotechnologies, Madison, WI) according to the manufacturer’s protocol. The gene expression profiling study, including labeling, hybridization, scanning, normalization, and data analysis, was performed by KangChen Bio-tech (Shanghai, China) using a Human Genome Oligo Microarray (4x44K, Agilent Technologies, Palo Alto, CA).

### Human corneal endothelial cells (HCECs) culture and treatment

Five corneal rims samples from normal donor corneas (three for 22 years old and two for 25 years old) were used to the HCECs culture and cell culture was performed by Wuhan PriCells Biomedical Technology Co., Ltd. (Wuhan, China) according to described protocols [8]. Briefly, Descemet’s membrane with intact endothelium was carefully dissected in small strips and then incubated in OptiMEM-I supplemented with 10 % FBS overnight to stabilize the cells before culture. After centrifugation, the strips were incubated in 0.02 % EDTA solution at 37 °C for 1 hour and cells were resuspended in culture medium containing OptiMEM-I, 8 % FBS, 5 ng/mL EGF, 20 ng/mL NGF, 100 g/mL pituitary extract, 20 g/mL ascorbic acid, 200 mg/L calcium chloride, 0.08 % chondroitin sulfate, 50 g/mL gentamicin, and antibiotic/antimycotic solution diluted 1/100. Cultures were then incubated at 37 °C in a 5 % carbon dioxide, humidified atmosphere.

The cultured HCECs were then subjected to various H_2_O_2_ concentrations (0 to 100 μM) for variable time periods (0 minutes, 1 hours, 2 hours and 6 hours) at 37 °C. The cells with no H_2_O_2_ treatment were used as controls. In addition, HCECs were also treated with SB203580 (10 μM) (Cell Signaling Technology, Inc., Danvers, MA) and the cells with no SB203580 treatment were used as controls.

### Senescence-associated β-galactosidase (SA-β-Gal) activity staining

Following the methods of previous reports [24, 25], human corneal whole mounts were fixed with 4 % formaldehyde (with the endothelial cell side up). The tissues were then incubated at 37 °C overnight using a senescence β-galactosidase staining kit (Beyotime Institute of Biotechnology, Shanghai, China) according to the manufacturer’s instructions. The staining was visualized and captured using a microscope that was equipped with a digital camera (Eclipse e800; Nikon).

### Immunofluorescent staining

For the immunofluorescent staining, mice corneal tissues were placed with the endothelium side up and fixed with 4 % PFA solution. Then, the samples were incubated overnight at 4 °C with the primary antibodies (p-ASK1 and p-p38). After washing with PBS, the samples were incubated for 1 h with FITC conjugated secondary antibody (1:100; Santa Cruz). The stained cells were counterstained with DAPI and viewed under an Eclipse TE2000-U microscope (Nikon, Tokyo, Japan).

### Statistical analysis

All results are expressed as the means ± SDs. The statistical analyses were performed using SPSS 15.0 software (SPSS, Chicago, IL). For the PCR Array and gene expression profiling study, an independent-Samples *t*-test was performed comparing the two different groups using the Kolmogorov–Smirnov test. For the analysis of the PCR and Western blot results, a One-Way analysis of variance (ANOVA) was performed to compare the groups using the Student–Newman–Keuls test and the Least Significant Difference procedure. *P*- values of less than 0.05 were considered to be statistically significant.

Information on the Real time PCR, Intracellular reactive oxygen species (ROS) measurement and the detection of mitochondrial ROS, Western blot analysis, and gene expression profiling studies available in the Additional file 1: Materials and Methods.

## Results

### Premature senescence in the endothelia of corneal allografts following PKP

We assessed premature senescence of endothelia using a normal-risk orthotopic mice corneal transplantation model for three times (totally 60 mice). We collected 8, 11, and 10 corneal grafts for these three times. For each time, four corneal grafts from each of the two groups were used for the detection of staining. That is, one corneal graft was separated into three pieces, and one pieces were used for the staining of the corneal endothelium with trypan blue and alizarin red S. The others were used for staining of SA-β-Gal and immunohistochemical analysis of 8-OHdG.

**Fig. 1:**
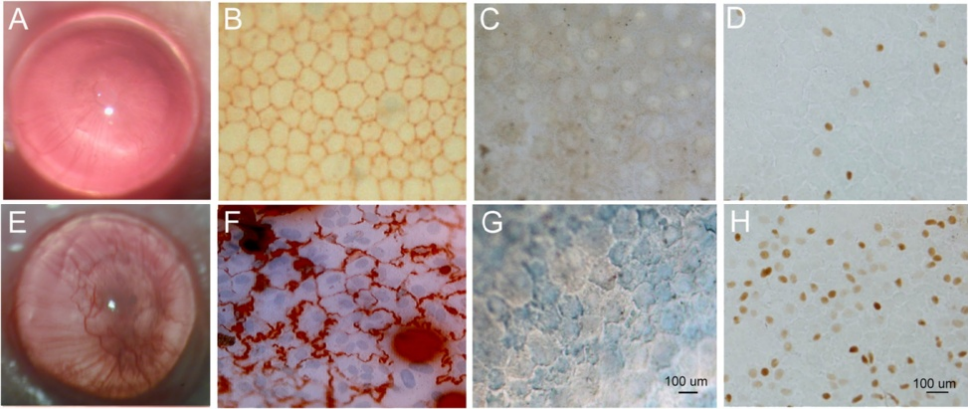
Premature senescence in mice endothelium of corneal dysfunctional allografts after PKP **a** Clinical evaluation of mouse corneal graft in syngeneic group (after 4.5 months post grafting); **b** Evaluation of endothelium of corneal graft in syngeneic group (*n* = 4); **c** Representative results of SA-β-Gal staining on corneal endothelium in syngeneic group; **d** Representative results of 8-hydroxydeoxyguanosine (8-OHdG) staining on corneal endothelium in syngenic group; **e** Clinical evaluation of mouse corneal graft in allogenic group (after 4.5 months post grafting); **f** Evaluation of endothelium of corneal graft in allogenic group; **g** Representative results of SA-β-Gal staining on corneal endotheliumin in allogenic group; **h** Representative results of 8-OHdG staining on corneal endothelium in allogenic group. Rejection of corneal grafts was observed in allogenic group as opacification of the cornea and new vessel in growth, compared with corneal grafts in syngeneic group **e**. In syngeneic group, the original hexagonal structure was maintained **b** compared with corneal grafts in allogenic group **f**. The endothelial cell borders of corneal grafts in allogeneic group were not clear, and polykaryocytes were observed in corneal endothelium of allogeneic group **f**. Compared with corneal grafts in allogeneic group **g**, SA-β-Gal positive cells were not observed on corneal endotheliumin in syngeneic group **c**. This revealed that dysfunctional corneal allografts exhibited characteristics of premature endothelial senescence. Compared with corneal grafts in syngenic group **d**, the strength and numbers of positive cells of 8-OHdG staining were less than that in allogenic group **h**

The results of the clinical evaluation of mouse corneal graft and corneal endothelium are shown in Fig. 1a and e. The original hexagonal structure of endothelial cells was maintained in the syngeneic group (Fig. 1b). The endothelial cell borders of the corneal grafts from the allogeneic group were opaque, and polykaryocytes were observed in corneal endothelium of the allogeneic group (Fig. 1f). SA-β-Gal-positive cells were observed in endothelium of the corneal grafts in allogeneic group (Fig. 1g), whereas SA-β-Gal-positive cells were not observed in endothelium of the corneal grafts in syngeneic group (Fig. 1c). Higher 8-OHdG expression was observed in the nuclei compared to the cytoplasm of the corneal endothelial cells. The percentage 8-OHdG expression was higher in the corneal graft nuclei of allogeneic group (Fig. 1d) than that of syngeneic group (Fig. 1h).

**Fig. 2:**
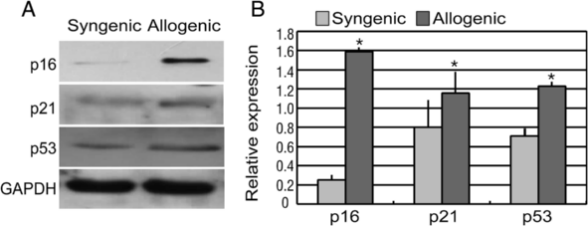
Up-regulated expression of p16^INK4A^, p21^Cip1^ and p53 proteins in mice endothelium of dysfunctional corneal allografts Expression of cell senescence related proteins, p16^INK4A^ , p21^Cip1/CDKN1A^ and p53 in mice endothelium of dysfunctional corneal allografts. Changes in protein expression as determined by Western blot. **a** data from the gels; **b** normalization to GAPDH. For each sample, the relative abundance of the protein of interest is determined by calculating the ratio of the intensity of the signal for the protein of interest to that of the normalization control GAPDH. Band densities determined by ImageJ software and compared with syngenic group. Expression of p16^INK4A^, p21^Cip1/CDKN1A^ and p53 were higher in the corneal endothelium of allogenic group than in the syngenic group (*t*-test, *P* < 0.05, n = 3). Significant differences between the corneal endothelium tissue in syngenic and allogenic groups are indicated by an asterisk (*P < 0.05)

We next compared the expression of p16^INK4A^, p21^WAF1/CIP1^ and p53 proteins in the endothelium of corneal grafts by western blot analysis (Fig. 2). A significant up-regulation of p16^INK4A^, p21^WAF1/CIP1^, and p53 protein expression was observed in the corneal endothelium of allogeneic grafts compared with syngeneic grafts.

**Fig. 3:**
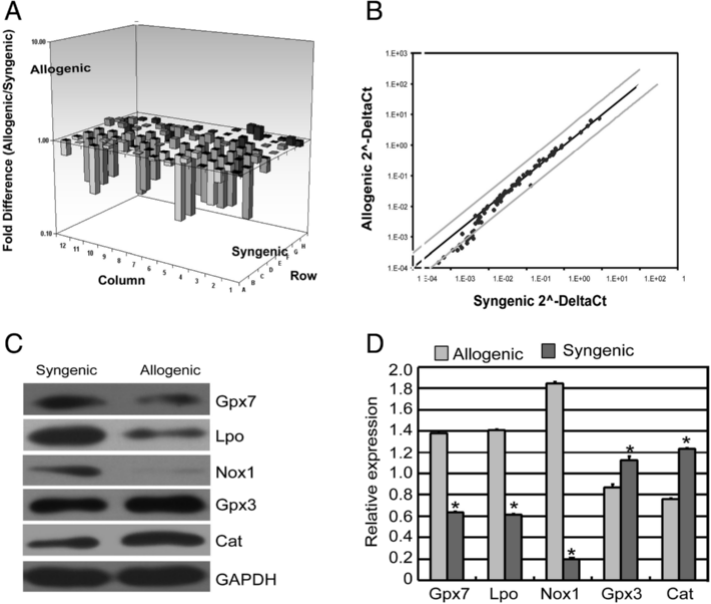
Oxidant-Antioxidant Imbalance in Mice Corneal Endothelium of Dysfunctional Allografts **a** Three-dimensional profile of Mouse Oxidative Stress and Antioxidant Defense RT^2^-PCR Arrays. **b** Scatter plot of expression differences among genes related to the oxidative stress and antioxidant defense. **c** and **d** Semiquantitative Western blot analyses were conducted to determine the relative protein level of the five genes found by PCR array analysis to be expressed at significantly different levels in corneal endothelium of allogenic and syngenic corneal grafts. Fig. 3 **c** shows representative blots for each down-expressed protein and Fig. 3 **d** shows representative blots for each up-expressed protein and the corresponding GAPDH bands. The syngenic group was used as control. For each sample, the relative abundance of the protein of interest is determined by calculating the ratio of the intensity of the signal for the protein of interest to that of the normalization control GAPDH. Band densities determined by ImageJ software and compared with syngenic group. Three additional experiments achieved equivalent results. Data are means ± SD (n = 3). All means marked with *(*t*-test, *P* < 0.05) are significantly different from the control

Then, the Mouse Oxidative Stress and Antioxidant Defense RT^2^-PCR Arrays were used to investigate the oxidant-antioxidant imbalance in the endothelium of corneal allografts after PKP. As indicated, a more than two fold change in expression, with *p* < 0.05, was considered to be statistically significant. Of the 84 genes assayed, 27 transcripts (32 %) were down-regulated; of these, 17 were expressed at more than two fold lower levels and 11 (13 %) were more highly expressed, as is shown in the three-dimensional profile (Fig. 3a) and the scatter plot (Fig. 3b). Of the genes that were expressed at lower levels, statistical significance was noted for 23 antioxidant genes (Table 3). The information for the more highly expressed genes is given in Table 4. The genes encoding Glutathione peroxidase 7 (Gpx7), Lactoperoxidase (Lpo) and NADPH oxidase 1 (Nox1) were expressed at 4.48-fold, 4.32-fold and 4.14-fold lower levels, respectively, in allogenic corneal endothelium compared with syngenic corneal endothelium.

**Table 3:** Genes down-regulated in endothelium of mice dysfunctional corneal allografts relative to syngenic control as detected by PCR array

Gene description	Symbol	Fold regulation	*P*
Antioxidant			
Glutathione Peroxidases (Gpx)			
Glutathione peroxidase 5	Gpx5	−3.18	0.1479
Glutathione peroxidase 6	Gpx6	−3.05	0.0021
Glutathione peroxidase 7	Gpx7	−4.48	0.0017
Peroxiredoxins (TPx)			
Peroxiredoxin 4	Prdx4	−1.22	0.0478
Other Peroxidases			
Lactoperoxidase	Lpo	−4.32	0.0007
Prostaglandin-endoperoxide synthase 2	Ptgs2	−1.43	0.0011
Recombination activating gene 2	Rag2	−3.00	0.0058
Thyroid peroxidase	Tpo	−2.64	0.0016
Other Antioxidants			
Nucleoredoxin	Nxn	−1.19	0.0105
ROS Metabolism			
Superoxide Metabolism			
Cytochrome b-245, alpha polypeptide	Cyba	−1.43	0.0122
Neutrophil cytosolic factor 2	Ncf2	−1.87	0.0045
NADPH oxidase 1	Nox1	−4.14	0.0052
NADPH oxidase 4	Nox4	−1.58	0.022
NADPH oxidase activator 1	Noxa1	−3.11	0.1457
NADPH oxidase organizer 1	Noxo1	−1.63	0.0349
RecQ protein-like 4	Recq14	−1.83	0.0016
Other genes involved in ROS Metabolism			
Interleukin 19	Il19	−3.37	0.0001
Interleukin 22	Il22	−3.41	0.0008
Oxidative stress responsive genes			
Dual oxidase 1	Duox1	−2.65	1.80E-05
Eosinophil peroxidase	Epx	−4.46	2.00E-04
Myeloperoxidase	Mpo	−3.49	0.0041
Membrane protein, palmitoylated 4 (MAGUK p55 subfamily member 4)	Mpp4	−3.11	0.0043
Nudix (nucleoside diphosphate linked moiety X)-type motif 15	Nudt15	−1.18	0.0106
Uncoupling protein 3 (mitochondrial, proton carrier)	Ucp3	−1.58	0.0088
Oxygen transporters			
Hemoglobin, theta 1A	Hbq1a	−3.13	0.0511
Myoglobin	Mb	−3.44	0.0604
Xin actin-binding repeat containing 1	Xirp1	−3.13	0.0003

**Table 4:** Genes up-regulated in endothelium of mice dysfunctional corneal allografts relative to syngenic control as detected by PCR array

Gene description	Symbol	Fold regulation	*P*
Antioxidant			
Glutathione Peroxidases (Gpx)			
Glutathione peroxidase 3	Gpx3	1.17	0.0118
Other Peroxidases			
Glutathione reductase	Gsr	1.25	0.0079
Catalase	Cat	1.24	0.0033
Adenomatosis polyposis coli	Apc	1.11	0.0144
Peroxiredoxin 6, pseudogene 1	Prdx6-ps1	1.17	0.0051
Tropomodulin 1	Tmod1	1.37	0.0004
ROS Metabolism			
Superoxide Metabolism			
Stearoyl-Coenzyme A desaturase 1	Scd1	1.09	0.0385
Oxidative stress responsive genes			
Isocitrate dehydrogenase 1 (NADP+), soluble	Idh1	1.19	0.0016
Protein phosphatase 1, regulatory (inhibitor) subunit 15b	Ppp1r15b	1.22	0.0048
Peroxiredoxin 2	Prdx2	1.29	0.0114
Oxygen transporters			
Solute carrier family 38, member 1	Slc38a1	1.11	0.0462

**Fig. 4:**
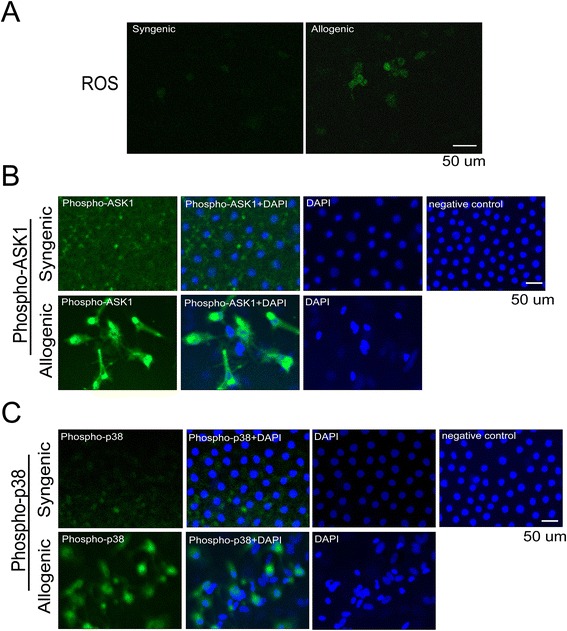
ROS accumulation and activation of ASK1/p38 signal pathway in Mice Corneal Endothelium of Dysfunctional Allografts **a** Representative results of ROS staining on corneal endothelium in syngenic group and allogenic group; **b** Up-regulation of phospho-ASK1 on corneal endothelium in syngenic group and allogenic group by immunofluorescence detection; **c** Up-regulation of phospho-p38 on corneal endothelium in syngenic group and allogenic group by immunofluorescence detection. Samples of corneal endothelium in syngenic group were used as the control group. 2 corneal grafts from each of the two groups were used for the detection of staining. That is, one corneal graft was separated into two pieces, and one piece was used for the staining of phospho-ASK1. The other was used for staining of phospho-p38

In the endothelium of allogenic corneal samples, Gpx7, Lpo, and Nox1 were expressed at 2.22-fold, 2.38-fold and 9.10-fold lower levels, respectively, relative to the endothelium in syngenic corneal samples by Western blot analyses (Fig. 3). In the endothelium of allogenic corneal samples, Gpx3 and Cat were expressed at 1.17-fold and 1.58-fold higher levels, respectively, than in the endothelium in the syngenic corneal samples. Then, we showed the results of the ROS accumulation and activation of ASK1/p38 signal pathway in mouse model in Fig. 4. The elevated ROS levels may be the results of the oxidant-antioxidant imbalance.

**Fig. 5:**
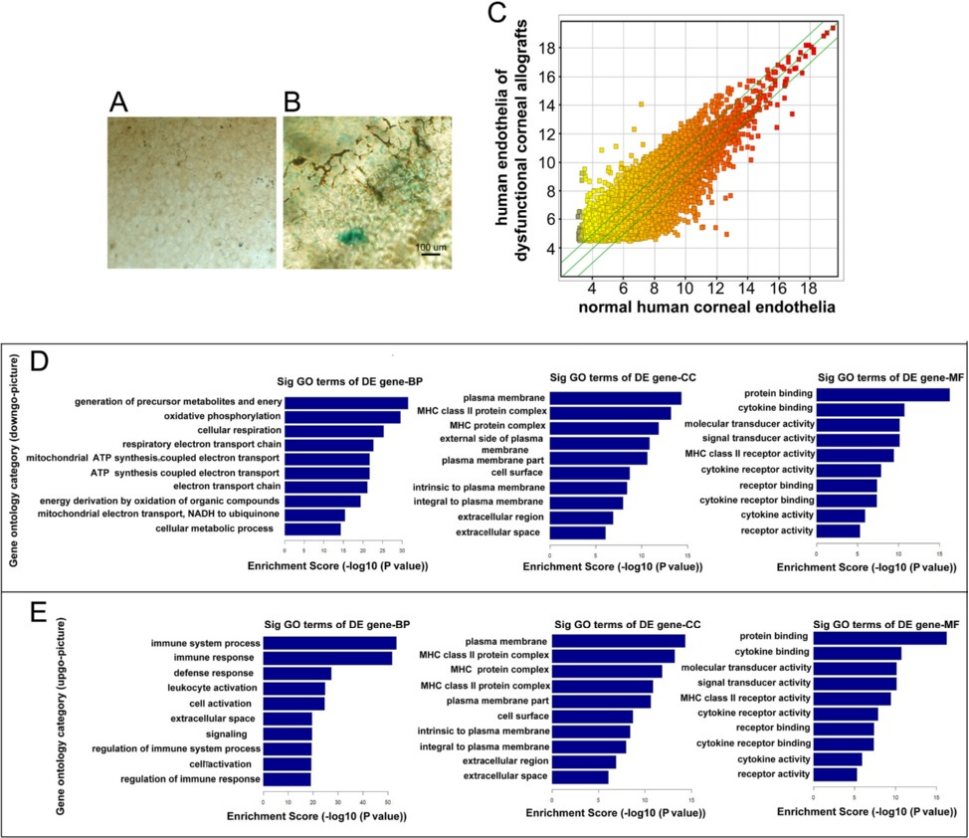
SA-β-Gal staining, gene expression profiling and microarray-based GO analysis of differentially expressed genes in human endothelium of dysfunctional corneal allografts Representative results of SA-β-Gal staining on human corneal endothelium in normal human corneal endothelium **a** and in dysfunctional allografts **b**. Compared with normal human corneal endothelium, SA-β-Gal positive cells were observed on human corneal endothelium in dysfunctional allografts. These results were consisted with that in the mice model. The result of gene expression profiling was shown as the scatter plot in Fig. 5-**c**. The scatter plot is a visualization that is useful for assessing the variation between human corneal endothelium in dysfunctional allografts (Y-axis) and in normal human corneal endothelium (X-axis). Microarray-based GO analysis of differentially expressed genes on human corneal endothelium between dysfunctional allografts and normal human corneal endothelium were shown in Fig. 5-**d** (the most significantly down-regulated genes) and Fig. 5-**e** (the most significantly up-regulated genes)

Representative results of SA-β-Gal staining on human corneal allografts and in normal human corneal endothelium were shown in Fig. 5-a and b. Compared with normal human corneal endothelium, more SA-β-Gal positive cells were observed on corneal endothelium in corneal allograft after PKP. These results were consistent with that in the mice model. The result of gene expression profilling was shown as the scatterplot in Fig. 5-c. The scatterplot is a visualization that is useful for assessing the variation between endothelium in corneal allografts (Y-axis) and in normal human corneal endothelium (X-axis). Microarray-based GO analysis of differentially expressed genes on endothelium between corneal allograft after PKP and normal human corneal endothelium was shown in Fig. 5-d (the most significantly down-regulated genes) and Fig. 5-e (the most significantly up-regulated genes). The three GO classifications, molecular function (MF), biological process (BP), and cellular component (CC), were evaluated separately and the significant terms of all ontologies are shown.

### Oxidative stress, elevated ROS levels, the up-regulation of CDK inhibitors and ROS-mediated p16 ^INK4A^ up-regulation in HCECs occur via the ASK1-p38 MAPK pathway

**Fig. 6:**
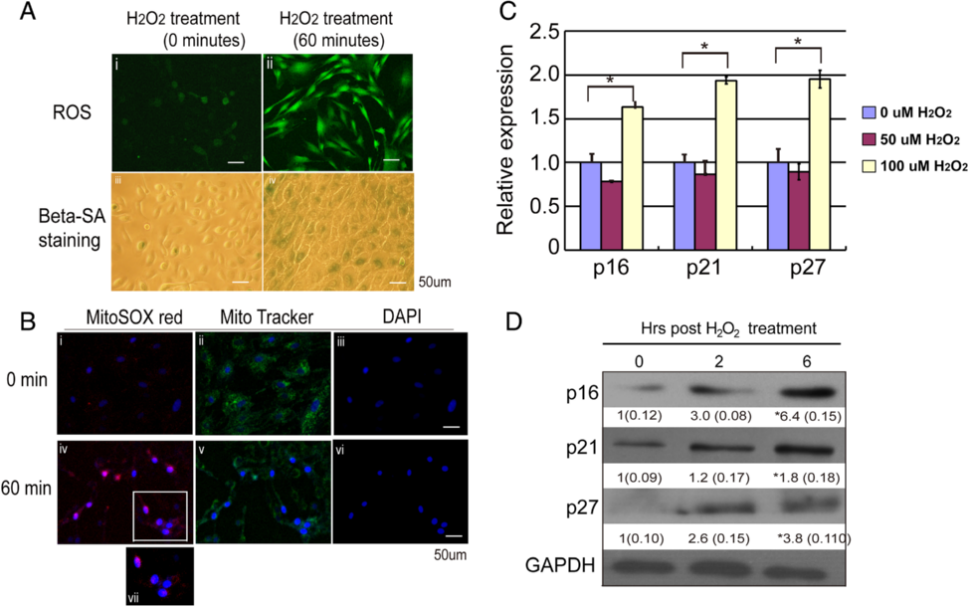
The effect of oxidative stress in cultured primary human corneal endothelial cells (HCECs) senescence *in vitro* **a** Oxidative stress on HCECs shows elevated ROS Levels. Intracellular ROS and mitochondria ROS accumulation by DCFH-DA staining after treatment of H_2_O_2_ using confocal microscopy. At 60 minutes after treatment with 100 μM H_2_O_2_, ROS generation was observed in HCECs (Fig. 6 **a**-i and -ii). SA-β-Gal positivity was also observed in HCECs after 60 minutes of treatment with 100 μM H_2_O_2_ (Fig. 6 **a**-iii and iv) compared with no H_2_O_2_-treatment. **b** Localization of MitoTracker Green FM with MitoSOX red in HCECs. Because of the localization of Mito Tracker Green (Fig. 6b-ii and -v), H_2_O_2_ treated cells showed red fluorescence in mitochondria, indicating increased mitochondrial ROS production (Fig. 6 **b**-iv), compared with control HCECs (Fig. 6b-i). **c** and **d** Oxidative stress on HCECs shows up-regulated Levels of CDK inhibitors. At 2 hours after 100 μM H_2_O_2_ treatment, a brief up-regulation of p16^INK4A^, p21^Cip1^, and p27^kip1^ mRNA was found in HCECs, whereas after 50 μM H_2_O_2_ treatment, the mRNA expressions of p16^INK4A^, p21^cip1^, and p27^kip1^ had no statistically difference between H_2_O_2_ treatment and no H_2_O_2_ treatment (Fig. 6 **c**). When HCECs were exposed to 100 μM H_2_O_2_, the level of p16^INK4A^, p21^cip1^, and p27^kip1^ protein expression was further elevated, and this up-regulation persisted from 2 to 6 hours post H_2_O_2_ treatment (Fig. 6 **d**). The HCECs with no H_2_O_2_ treatment was used as control. Three more additional experiments achieved equivalent results. Data are means ± SD (*n* = 3). All means marked with * (*P* < 0.05) are significantly different from the control

Given that the oxidant-antioxidant imbalance was involved in endothelium of corneal allografts, we developed an *in vitro* experimental model using H_2_O_2_ treatment to simulate a state of oxidative stress. Primary culture of human corneal endothelial cells with generic function associated markers such as Na^+^K^+^ATPase and ZO-1 as well as PRDX6 were characterized. Intracellular ROS and mitochondria ROS accumulation were compared. ROS generation was observed in HCECs 60 minutes following treatment with 100 μM H_2_O_2_ (Fig. 6a-i and 6a -ii). The data indicate that intracellular ROS levels are much higher in HCECs following H_2_O_2_ treatment than in untreated cells. SA-β-Gal positivity was also observed in HCECs after 60 minutes of treatment with 100 μM H_2_O_2_ (Fig. 6a-iii and 6a-iv). To determine whether ROS production is enhanced in the mitochondria of HCECs following H_2_O_2_ treatment, the localization of MitoTracker Green FM with MitoSOX red was performed. As revealed by the localization of Mito Tracker Green (Fig. 6b-ii and 6a-v), H_2_O_2_ treated cells exhibited red fluorescence in mitochondria, indicating increased mitochondrial ROS production (Fig. 6b-iv) compared with the control HCECs (Fig. 6b-i).

Inhibitors of cyclin-dependent kinases (CDKs) are considered to play critical roles in cell cycle arrest and premature senescence [8, 26]. We therefore investigated the effects of ROS on the levels of CDK inhibitors in HCECs, including p16^INK4A^, p21^cip1^, and p27^kip1^. At 2 hours after 100 μM H_2_O_2_ treatment, a brief up-regulation of p16^INK4A^, p21^cip1^, and p27^kip1^ mRNA was observed in HCECs (Fig. 6c). We also detected the protein expression levels of p16^INK4A^, p21^cip1^, and p27^kip1^ by western blot analysis, as is shown in Fig. 6d. When the HCECs were exposed to 100 μM H_2_O_2_, the levels of p16^INK4A^, p21^cip1^, and p27^kip1^ protein expression were elevated, and this up-regulation persisted from 2 to 6 hours post-H_2_O_2_ treatment.

**Fig. 7:**
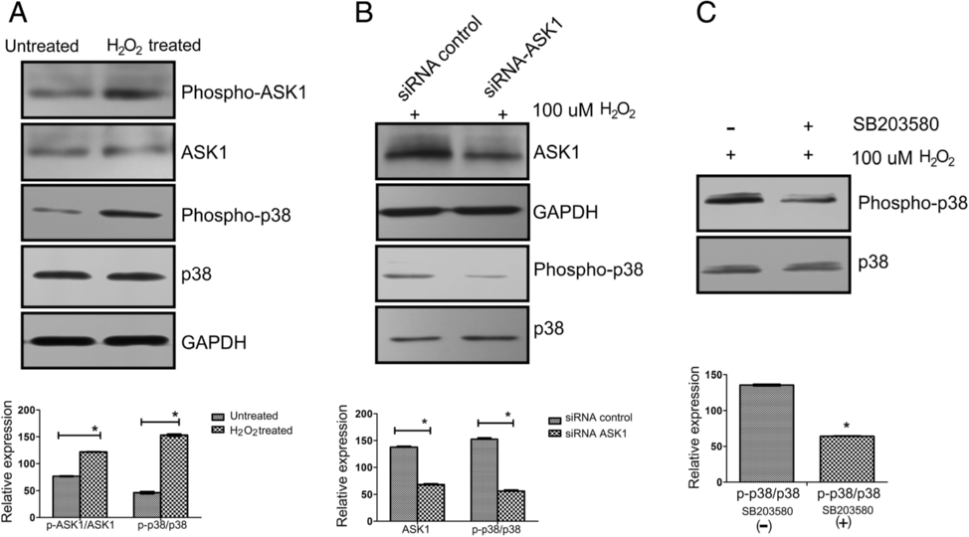
ASK1/p38 signaling is activated in cultured HCECs *in vitro*. **a** The protein level of ASK1 or phosphorylated ASK1 was measured by Western blot and then the activation of p38 MAPK was also compared with or without H_2_O_2_ treatment of HCECs *in vitro*. Top panel: shows the representative data from the gels; bottom panel: the results normalized to GAPDH. GAPDH served as the loading control. The HCECs with no H_2_O_2_ treatment was used as control. Three more additional experiments achieved equivalent results. Data are means ± SD (*n* = 3). All means marked with * (*t*-test, *P* < 0.05) are significantly different from the control. **b** siRNA ASK1 decreased H_2_O_2_-induced p38 activation. HCECs were transfected with 150 nM ASK1 siRNA and control siRNA, and 24 h later, cells were treated with 100 μM H_2_O_2_ for 4 h. The protein expression was measured by Western Blot. Top panel: shows the representative data from the gels; bottom panel: the results normalized to GAPDH. GAPDH served as the loading control. Three more additional experiments achieved equivalent results. Data are means ± SD (*n* = 3). All means marked with * (*P* < 0.05) are significantly different from the control. **c** p38 signaling is required for the response to ROS in cultured HCECs *in vitro*. HCECs were pretreated with or without SB203580 (10 μM) for 2 h and then coincubated with 100 μM H_2_O_2_ for 4 h. The protein levels were measured by Western blot assays. Top panel: shows the representative data from the gels; bottom panel: the results normalized to GAPDH. GAPDH served as the loading control. HCECs treated without SB203580 was used as control. Three more additional experiments achieved equivalent results. Data are means ± SD (*n* = 3). All means marked with * (*P* < 0.05) are significantly different from the control

To address whether H_2_O_2_-induced HCECs senescence is related to the activity of ASK1-p38 MAPK pathway, the protein levels of ASK1 or phosphorylated ASK1 was measured by Western blot analysis. p38 MAPK activation was then compared between the H_2_O_2_-treated and untreated HCECs *in vitro*. Fig. 7a presents representative the results of the Western blot studies. The phosphorylation levels of ASK1 and p38 MAPK significantly increased in HCECs following H_2_O_2_ treatment. We also used siRNA that specifically silences ASK1 and SB203580, a widely used p38 inhibitor, to investigate the molecular mechanisms that underlie H_2_O_2_-induced endothelial cell senescence in cultured HCECs. By Western blotting analysis, we found that the expression of ASK1 was down-regulated after transfection with ASK1-siRNA and ASK1-siRNA also decreased the activation of MAKP in HCECs (Fig. 7b). By the pharmacological inhibitor SB203580, the expression of p38 was down-regulated in HCECs treated with H_2_O_2_ (Fig. 7c).

**Fig. 8:**
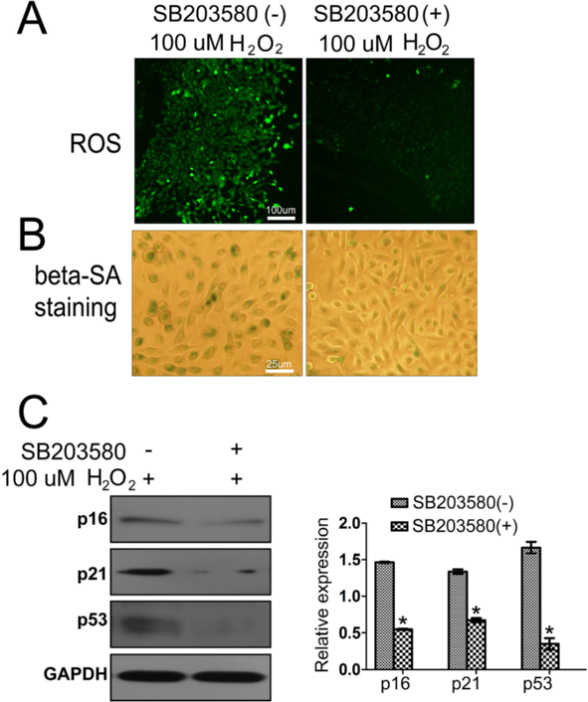
ASK1/p38 signaling is required for the response to ROS in cultured HCECs *in vitro*. **a** The level of ROS was measured and compared with or without SB203580 treatment of HCECs under H_2_O_2_ induced oxidative stress conditions *in vitro*. Bar = 100 μm. The HCECs with no SB203580 treatment was used as control. Three more additional experiments achieved equivalent results. **b** SA-beta-Gal staining was performed and compared with or without SB203580 treatment of HCECs under H_2_O_2_ induced oxidative stress conditions *in vitro*. Bar = 25 μm. The HCECs with no SB203580 treatment was used as control. Three more additional experiments achieved equivalent results. **c** The protein level of p16, p21 and p53 was measured by Western blot and compared with or without SB203580 treatment of HCECs *in vitro*. Left panel: shows the representative data from the gels; right panel: the results normalized to GAPDH. GAPDH served as the loading control. The HCECs with no SB203580 treatment was used as control. Three more additional experiments achieved equivalent results. Data are means ± SD (*n* = 3). All means marked with *(*P* < 0.05) are significantly different from the control

To address whether p38 signaling is required for senescence in response to ROS accumulation in HCECs, we showed the data of the effect of SB203580 on cell senescence in Fig. 8. We found that the ROS levels and the strength of SA-β-Gal staining were decreased after SB203580 treatment (Fig. 8-a and 8-b). We also detected the expression of cell senescence related proteins, including p16^INK4A^, p27^kip1^ and p53. As shown in Fig. 8-c, the expression of the three proteins was down-regulated after treatment with SB203580 in HCECs, compared with no treatment of SB203580. These results imply that the ASK1-p38 MAPK pathway may be involved in ROS-induced CECs senescence.

## Discussion

In this study, we report a studying on penetrating keratoplasty and in particular the potential mechanisms behind post-operative failure (for example, corneal opacity and the role of the endothelium). We use a combination of descriptive data from human PKP samples, together with a mouse model (syngeneic versus allogeneic corneal transplantations) and some cell work *in vitro* with human HCECs treated with peroxide.

In the context of PKP, premature senescence is important clinically not only because aging alters corneal function but also because old corneas perform poorly when transplanted. The endothelium is a major determinant of graft survival. Since stress might accelerate ageing changes, it will be instructive to understand the mechanisms of premature senescence in HECEs. HCECs are arrested in the G1 phase of the cell cycle and given the correct culture medium, HCECs can be grown for many population doublings in culture [27]. We previously reported an age-related increase in p16^INK4A^ expression in normal HCECs *in vivo* [28] and in the senescence accelerated mouse (SAM), indicating that the increased expression of p16^INK4A^ is an age-dependent phenomenon in the corneal endothelium [29]. We also observed that the high expression of p16^INK4A^ and low expression of Bmi1 are associated with cellular senescence of HCECs [30]. Other groups also investigate the characterisation of cellular senescence mechanisms in HCECs [27]. The above studies reported the common aging phenotype and molecular mechansims of normal HCECs.

The accumulation of SA-β-Gal is suggested to be a specific marker of cell senescence [31, 32] and 8-OHdG is the most frequently detected and studied biomarker of ROS for cancer, atherosclerosis and diabetes [33]. In the present study, we found the elevated level of 8-OHdG, suggesting the existence of ROS in corneal endothelium after PKP. Furthermore, the expressions of p16^INK4a^, p21^WAF1/CIP1^ and p53 proteins in corneal endothelium of allogeneic grafts were correlated with the accumulation of SA-β-Gal and 8-OHdG, and their expressions were consistent with their induction as a function of cell aging.

ROS play a role in cellular functions including signal transduction at normal concentrations [34]. But an imbalance between generation of ROS and capacity of antioxidants to neutralize ROS can result in disruption of cellular redox status, leading to oxidative stress [35]. Several studies have suggested that low doses of H_2_O_2_ promote cell proliferation, whereas high levels of ROS can induce DNA damage and trigger cell aging, eventually causing cells to enter senescence prematurely [36, 37]. Thus, we developed an *in vitro* experimental model using treatment of H_2_O_2_ to simulate cells in a state of oxidative stress. In this study, the dose of H_2_O_2_ that is being used was 100 μM. Low concentrations of H_2_O_2_ (less than 10 μM) were found to stimulate cell proliferation in fibroblasts [38]. Whereas intermediate concentrations of H_2_O_2_ (10 ~ 150 μM) caused cell growth arrest and senescence [4, 39].

For PKP, the cumulative burden of injury may exhaust the ability of corneal endothelial cells to repair and remodel to maintain tissue integrity. PKP may represent a final common pathway that greatly accelerated by the special stresses on the transplant, such as aging, nonimmune injury, and rejection. This is more a result of endothelial deterioration for cornea. In our study, the data suggest a ROS/p38 driver of senescence in corneal syngeneic transplants. The reasons for such transplant generate ROS and rigger senescence may be age-related diseases before the transplant, peri-transplant injury or rejection. This is a key question both to understand the significance of our results and also to start to understand why some PKP cases fail. To test this properly would require a quite complex mouse interventional study (p38 inhibition during transplantation), we do attempt a cell line model system to test the causality. Using 100 μM peroxide we induce senescence in HCECs, and interestingly show that both ROS markers and SA-β-Gal elevation can be suppressed using a p38 small molecule inhibitor (Fig. 8). Although there are some obvious concerns regarding the very high peroxide levels we use, these interventional studies do give initial support for the model we suggest.

## Conclusions

In conclusion, our observations indicate an elevation of markers of ROS (both directly and via looking at oxidative defence gene expression), and markers of senescence (SA-β-Gal and proteins such as p16^INK4a^/p21^WAF1/CIP1^/p53 that are associated with senescence). We also show evidence for activation of the p38MAPK and ASK1 pathway in this situation. These observational data would be consistent with a pathway of ROS > p38/ASK1 > senescence. This in turn suggests a SIPS process taking place in the corneal syngeneic transplants. Our results will give new insights into the molecular pathogenesis of corneal allograft dysfunction, providing future targets for therapeutic intervention.

## Additional file

Additional file 1:
**Supplemental Materials and Methods.** (DOCX 20 kb)

## Abbreviations

HCEC: Human corneal endothelial cells
LKP: Lamellar keratoplasty
PKP: Penetrating keratoplasty
ROS: Reactive oxygen species
SIPS: Stress- induced prematural senescence
