# Supplementary material for: Oxidative stress and premature senescence in corneal endothelium following penetrating keratoplasty in an animal model
Source: BMC Ophthalmol. 2016 Feb 2;16:16. doi: 10.1186/s12886-016-0192-6 (PMC4736695; doi:10.1186/s12886-016-0192-6)
Supplement: Additional file 1: — Supplemental Materials and Methods. (DOCX 20 kb) [file 12886_2016_192_MOESM1_ESM.docx]

Title of data

Supplemental Materials and Methods

**Real Time PCR**

We used real time PCR to detect the mRNA expression levels of p16^INK4A^, p21^cip1^, and p27^kip1^ of HCECs following treatment with H_2_O_2_. The detection system (ABI Prism 7500 System; Applied Biosystems) was employed using real-time PCR, as recommended by the manufacturer. Each sample was assayed in duplicate (TaqMan Universal PCR Master Mix; Applied Biosystems). The primers and oligonucleotide probes that were used are listed in Supplemental Table 1. The quantification data were analyzed using SDS system software (7500 System; Applied Biosystems). The log-linear portion of the fluorescence versus cycle plot was extended to determine a fractional cycle number at which a threshold fluorescence was obtained (threshold cycle, Ct); this number was used as a reference for each analyzed gene and for GAPDH.

**Intracellular Reactive oxygen species (ROS) Measurement and the Detection of Mitochondrial ROS**

The levels of HCECs intracellular ROS and mitochondrial ROS were measured 60 minutes following H_2_O_2_ treatment. For intracellular ROS detection, the cells were mixed with 10 μM 2’, 7’-dichlorodihydrofluorescein diacetate (DCFH-DA; Molecular Probes, Eugene, OR, USA) in PBS (pH 7.4) for 45 minutes in the dark. The fluorescence levels were detected using Nikon confocal microscope system C1si (Nikon Corporation, Tokyo, Japan) using excitation and emission wavelengths of 488 and 510- 550 nm. For mitochondrial ROS detection, the cells were incubated with 5 μM MitoSOX Red and 1 μM MitoTracker Green FM (Molecular Probes, Eugene, OR, USA) at 37 °C for 15 minutes. Excess stains were removed, and the cells were imaged using the same Nikon confocal microscope system as above. The excitation and emission wavelengths for MitoTracker Green were 490 and 516 nm. For MitoSOX Red, the excitation and emission wavelengths were 560 and 600 nm. The nuclei were stained with 4’6-diamidino-2-phenylindole (DAPI; Sigma-Aldrich, Shanghai, China).

For ROS staining on mice corneal endothelium in syngenic group and allogenic group, corneal endothelial cells were harvested with 1X accutase (Cat#A6964, Sigma-Aldrich, Shanghai, China) and centrifuged at 1000 rpm for 5 minutes, washed with PBS, and fixed overnight in 4% paraformaldehyde (PFA) solution in PBS at 4 °C. The cells were attached to coated microscope slides in a TXT3 centrifuge (Lu Xiang Yi Centerfuge Instrument Co., Ltd, Shanghai, China) at 500 rpm for 5 min and dried overnight on a slide warmer at 37 °C. ROS staining was performed on the slides as described above.

**Western Blot Analysis**

The corneal endothelium samples were excised and homogenized in PBS and the supernatant was assayed using SDS-polyacrylamide gels (Mini-Protean II system; Bio-Rad Laboratories, Mississauga, ON, Canada) and blotted onto polyvinylidene difluoride membranes. The membranes were then incubated overnight with primary antibodies at 4°C overnight. Next, the membranes were stained with specific secondary antibodies and the hybridized bands were detected with an enhanced chemiluminescence kit (PerkinElmer Life Sciences, Boston, MA). The information regarding the primary antibodies that were used for all of the western blot analyses is given in Supplemental Table 2.

**Gene Expression Profiling Study**

Total RNA from each sample was quantified using the NanoDrop ND-1000 and the RNA integrity was assessed using standard denaturing agarose gel electrophoresis. For microarray analysis, Agilent Array platform was employed. The sample preparation and microarray hybridization were performed based on the manufacturer’s standard protocols. Briefly, total RNA from each sample was amplified and transcribed into fluorescent cDNA with using the manufacturer’s Agilent’s Quick Amp Labeling protocol (version 5.7, Agilent Technologies). The labeled cDNAs were hybridized onto the Whole Human Genome Oligo Microarray (4x44K, Agilent Technologies, Palo Alto, CA). After having washed the slides, the arrays were scanned by the Agilent Scanner G2505C.

Agilent Feature Extraction software (version 11.0.1.1) was used to analyze acquired array images. Quantile normalization and subsequent data processing were performed using the GeneSpring GX v11.5.1 software package (Agilent Technologies, Palo Alto, CA). After quantile normalization of the raw data, genes that at least 2 out of 2 samples have flags in Detected (“All Targets Value”) were chosen for further data analysis. Differentially expressed genes were identified through Fold Change filtering. GO Analysis were applied to determine the roles of these differentially expressed genes played in these biological GO terms using the DAVID Bioinformatics Resources (<http://david.abcc.ncifcrf.gov>).

**Immunofluorescent staining**

For the immunofluorescent staining, mice corneal tissues were placed with the endothelium side up and fixed with 4% PFA solution. Then, the samples were incubated overnight at 4°C with the primary antibodies (p-ASK1 and p-p38). After washing with PBS, the samples were incubated for 1 h with FITC conjugated secondary antibody (1:100; Santa Cruz). The stained cells were counterstained with DAPI and viewed under an Eclipse TE2000-U microscope (Nikon, Tokyo, Japan).

**Supplemental Table 1. Primers used in real time PCR**

| **Gene Name** | **Primer Sequences** | **Probe Sequence** |
| --- | --- | --- |
| ***p16*** | F: cttcctggacacgctggt | fam-gacctggctgaggagctg-tamra |
| (NM_000077.4) | R: gcatggttactgcctctggt |  |
| ***p21*** | F: cag acc agc atg aca gat ttc | fam-acc act cca aac gcc ggc tg-tamra |
| (NM_000389.4) | R: tta ggg ctt cct ctt gga ga |  |
| ***p27*** | F: cct cct cca aga caa aca gc | fam-tcg agt tcc tga caa gcc acg c-tamra |
| (NM_004064.3) | R: cat tca gag cgg gat tat ctt t |  |

**Supplemental Table 2. Antibodies used in Western blot**

| **Antibody** | **Company** | **Cat. No** | **Isotype** | **Dilution** |
| --- | --- | --- | --- | --- |
| p16 | Santa Cruz^*^ | sc-1661 | mouse IgG | 1/200 |
| p21 | Abcam^†^ | ab92675 | Rabbit IgG | 1/1000 |
| p27 | Abcam | ab7961 | Rabbit IgG | 1/1000 |
| p53 | Abcam | ab8590 | Rabbit IgG | 1/1000 |
| Gpx7 | Santa Cruz | sc-160062 | Goat IgG | 1/200 |
| Gpx3 | Santa Cruz | sc-50496 | Rabbit IgG | 1/200 |
| Lpo | Abcam | ab82150 | Goat IgG | 1/500 |
| Nox1 | Abcam | ab78016 | Rabbit IgG | 1/200 |
| Cat | Abcam | ab1877 | Rabbit IgG | 1/1000 |
| p-ASK1 | Santa Cruz | sc-33362 | Rabbit IgG | 1/200 |
| ASK1 | Santa Cruz | sc-6368 | Rabbit IgG | 1/200 |
| Phospho-p38 | CST^‡^ | #9211 | Rabbit IgG | 1/1000 |
| p38 | CST | #9212 | Rabbit IgG | 1/1000 |

* Santa Cruz Biotechnology, Inc., Santa Cruz, CA.

† Abcam, Inc., Cambridge, MA.

‡ Cell Signaling Technology, Inc., Danvers, MA.
